# Effects of Diet, Aerobic Exercise, or Both on Non-HDL-C in Adults: A Meta-Analysis of Randomized Controlled Trials

**DOI:** 10.1155/2012/840935

**Published:** 2012-11-08

**Authors:** George A. Kelley, Kristi S. Kelley

**Affiliations:** Meta-Analytic Research Group, Department of Biostatistics, School of Public Health, Robert C. Byrd Health Sciences Center, West Virginia University, Morgantown, WV 26506-9190, USA

## Abstract

*Purpose*. To use the meta-analytic approach to examine the effects of diet (D), aerobic exercise (E), or both (DE) on non-high-density lipoprotein cholesterol (non-HDL-C) in adults. *Methods*. Randomized controlled trials in adults ≥18 years of age were included. A mixed-effect model was used to combine effect size (ES) results within each subgroup and to compare subgroups (*Q*_*b*_). Heterogeneity was examined using the *Q* and *I* ^2^ statistics, and 95% confidence intervals (CI) were also calculated. Statistical significance was set at *P* ≤ 0.05, while a trend for statistical significance was set between *P* > 0.05, and ≤0.10. *Results*. A statistically significant exercise minus control group decrease in non-HDL-C was found for DE (7 ESs, 389 participants, $\bar{x}=-11.1$ mg/dL, 95%  CI = −21.7 to −0.6, *P* = 0.04, *Q* = 2.4, *P* = 0.88, *I* ^2^ = 0%), a trend for the D group (7 ESs, 402 participants, $\bar{x}=-8.5$ mg/dL, 95%  CI = −18.6 to 1.6, *P* = 0.10, *Q* = 0.76, *P* = 0.99, *I* ^2^ = 0%), and no change for the E group (7 ESs, 387 participants, $\bar{x}=3.0$ mg/dL, 95% CI = −7.1 to 13.1, *P* = 0.56, *Q* = 0.78, *P* = 0.99, *I* ^2^ = 0%). Overall, no statistically significant between-group differences were found (*Q*_*b*_ = 4.1, *P* = 0.12). *Conclusions*. Diet combined with aerobic exercise may reduce non-HDL-C among adults in some settings.

## 1. Introduction

 Cardiovascular disease is a major public health problem affecting an estimated 82.6 million adults in the United States (USA) [1]. In terms of mortality, heart disease is the leading cause of death in the USA, affecting 616,628 people (25% of all deaths) in 2008 [2]. Not surprisingly, the economic costs associated with cardiovascular disease are also high. In 2008, the annual direct and indirect costs of cardiovascular disease in the USA were estimated to be $297.7 billion [1]. 

 Less than optimal levels of lipids and lipoproteins are a major risk factor for cardiovascular morbidity and mortality in adults [1]. According to recent estimates, 33.6 million USA adults have total cholesterol (TC) levels ≥240 mg/dL, 71.3 million have low-density-lipoprotein cholesterol (LDL-C) levels ≥130 mg/dL and 41.8 million have high-density lipoprotein cholesterol (HDL-C) levels ≤40 mg/dL [1]. Currently, the primary target of lipid-lowering therapy in adults is low-density lipoprotein cholesterol (LDL-C) with non-high-density lipoprotein cholesterol (non-HDL-C) recommended as a secondary target of therapy in adults with triglyceride levels ≥200 mg/dL [3]. However, it has been suggested that non-HDL-C may be a more relevant target for lipid-lowering therapy because it contains all the lipids and lipoproteins considered to be atherogenic (low-density lipoprotein cholesterol, lipoprotein (a), intermediate-density lipoprotein, very-low-density lipoprotein) [4, 5]. Indeed, previous meta-analytic research has shown that non-HDL-C is a better predictor than LDL-C for future cardiovascular risk. For example, Boekholdt et al. found that non-HDL-C was a better predictor than LDL-C for future risk of cardiovascular events in statin-treated patients [6]. In addition, another meta-analysis found that over a 10-year period, a focus on lowering non-HDL-C versus LDL-C would prevent 300,000 more fatal or nonfatal ischemic cardiovascular events [7]. From a practical perspective, the assessment of non-HDL-C versus LDL-C may be preferred because (1) it can be assessed in the nonfasting state, (2) incurs no additional costs because it is calculated as the difference between TC and HDL-C, and (3) has well-documented benefits [5]. 

Aerobic exercise and diet are first-line lifestyle interventions recommended for improving lipids and lipoproteins, including LDL-C, in adults [3]. Recently, aggregate data meta-analytic research of randomized controlled trials addressing the effects of diet (D), aerobic exercise (E), or both (DE) on lipids and lipoproteins in adults were reported by the authors [8]. Interventions had to last at least 4 weeks with diet including any type previously considered to improve lipids and lipoproteins in adults (low saturated fat, caloric restriction, etc.) [3]. For both the D and DE groups, statistically significant intervention minus control (C) group improvements were observed for TC, LDL-C, and triglycerides (TG), but not HDL-C. For the E groups, improvements were limited to TG. When between-group comparisons were conducted, reductions in TC and LDL-C were greater in both the D and DE groups versus E group (*P* < 0.05). No other between-group differences were observed. Unfortunately, none of the studies reported data for non-HDL-C, including dispersion data. In this brief paper, we use an existing method for estimating measures of dispersion for non-HDL-C based on data reported for TC and HDL-C [9] in order to conduct a meta-analysis on the effects of D, E, or both on non-HDL-C in adult humans. 

## 2. Methods 

### 2.1. Study Eligibility, Data Sources, Data Extraction, and Risk of Bias Assessment

Study eligibility, data sources, data extraction, and risk of bias assessment have been previously described in detail elsewhere [8]. Briefly, studies in any language were included if they were randomized controlled trials ≥4 weeks that included D, E, DE, and C groups in adults ≥18 years of age and in which mean and dispersion data for TC and HDL-C were available for calculating non-HDL-C. Data sources included searching nine electronic databases, cross-referencing, and expert review. Dual data extraction occurred using predeveloped codebooks. Risk of bias was assessed by both authors, independent of each other, using the Cochrane Risk of Bias Assessment instrument [10].

### 2.2. Statistical Analysis

#### 2.2.1. Calculation of Treatment Effects from Each Study

The primary outcome for this meta-analysis was treatment effect changes in non-HDL-C. First, each intervention (D, E, DE) and Control (C) group result was calculated as the change outcome difference in TC minus the change outcome difference in HDL-C. Second, the variance for non-HDL-C for each result from each group (D, E, DE, C) was calculated by pooling the variances of the change outcome differences for TC and HDL-C. Third, treatment effect changes in non-HDL-C were calculated as the intervention (D, E, DE) minus the C result. Variances for these changes were calculated by pooling intervention (D, E, DE) and C results [9]. 

#### 2.2.2. Pooling of Treatment of Effects

A mixed effects model was used to pool non-HDL-C treatment effects (intervention minus control) for each group (D, E, DE) from each study and to compare results across the three groups. This consisted of a random-effects model to combine studies within each group (D, E, DE) and a fixed-effect model to compare results between groups (*Q*_*b*_). Study-to-study variance (tau-squared) was not assumed to be equal for all subgroups. A *z*-score alpha value of ≤0.05 (two-tailed) was considered statistically significant while alpha values >0.05 but ≤0.10 were considered as a trend. Precision of treatment effects estimates for non-HDL-C was determined using two-tailed 95% confidence intervals (CIs) based on *z*. Estimation of treatment effects for non-HDL-C in a new trial was calculated using 95% prediction intervals (PI) [11–13]. Any statistically significant outliers (*P* ≤ 0.05) were deleted from the model. 

 Heterogeneity of results for each group was examined using the *Q* and *I* ^2^ statistics [14, 15]. The alpha value for statistical significance for *Q* was set at *P* ≤ 0.10. For *I* ^2^, values of 25% to <50% were considered small, 50% to <75% medium, and ≥75% large [15]. Potential bias due to small-study effects was examined using a funnel plot along with the data imputation approach of Duval and Tweedie [16, 17]. Simple, mixed-effects meta-regression was conducted to examine the effects of age, baseline non-HDL-C, and intervention minus control group changes in body weight on changes in non-HDL-C in each group (D, E, DE). A two-tailed alpha value of ≤0.05 was considered as a statistically significant association while alpha values >0.05 and ≤0.10 were considered as a trend.

All data were analyzed using Comprehensive Meta-Analysis (version 2.2) [18], Microsoft Excel 2007 [19], and SSC-Stat (version 2.18) [20].

## 3. Results

Six studies representing 788 men and women (D = 207, E = 192, DE = 194, C = 195) from 28 groups (7 groups each for D, E, DE, and C) met all eligibility criteria [21–26] and have been described in detail elsewhere [8]. The baseline between-study range for all groups combined was 34 to 57 years for age ($\overset{-}{x}\pm \text{SD}=46.5\pm 6.5$ years), 63 to 100 kg for bodyweight ($\overset{-}{x}\pm \text{SD}=80.8\pm 13.4$ kg), 180 to 254 mg/dL for TC ($\overset{-}{x}\pm \text{SD}=213.6\pm 22.0$ mg/dL), and 36 to 63 mg/dL for HDL-C ($\overset{-}{x}\pm \text{SD}=48.3\pm 7.7$ mg/dL). 

**Figure 1:**
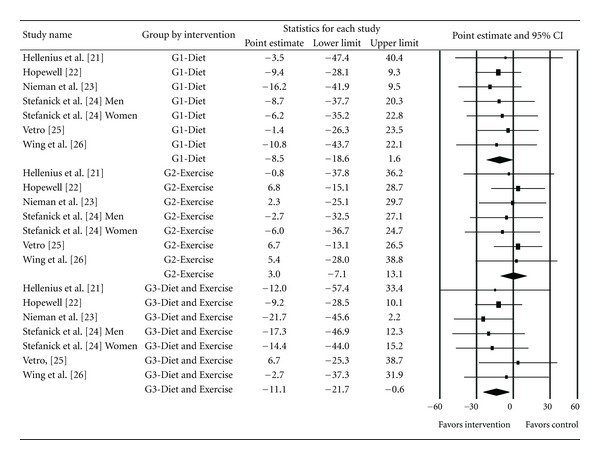
Forest plot for intervention minus control group changes in non-HDL-C according to Diet, Exercise and Diet and Exercise interventions. The black squares for each result represent the difference in non-HDL-C in mg/dL while the left and right extremes of the squares represent the corresponding 95% confidence intervals. The middle of the black diamond for the three groups represent the overall mean difference while the left and right extremes of the diamonds represent the corresponding 95% confidence intervals based on a random-effects model. To convert mg/dL to mmol, divide by 38.67.

**Figure 2:**
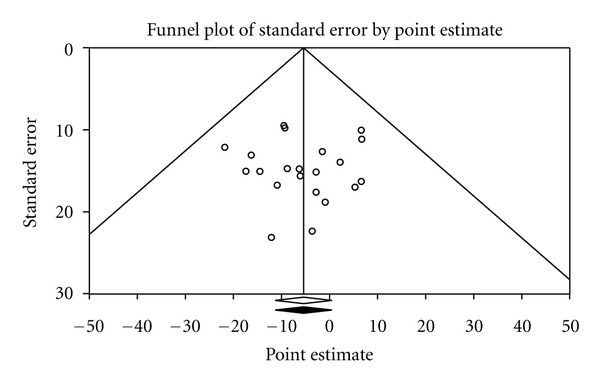
Funnel plot for intervention minus control group changes in non-HDL-C across all results. The *x*-axis represents changes in non-HDL-C in mg/dL, while the *y*-axis represents the standard error of the changes in non-HDL-C in mg/dL. The middle of the hollow diamond represents the original overall mean difference, while the left and right extremes of the diamond represent the corresponding 95% confidence intervals based on a random-effects model. The middle of the black solid diamond represents the mean difference in non-HDL-C, adjusted for small-study effects, while the left and right extremes of the diamond represent the corresponding 95% confidence intervals based on a random-effects model. As can be seen by the matching diamonds, no adjustment (imputation) was necessary. To convert mg/dL to mmol, divide by 38.67.

 Baseline values for non-HDL-C are shown in Table 1, group changes in Table 2, and study-level changes in (Figure 1). As can be seen, there was a statistically significant intervention minus control group decrease for non-HDL-C in the DE group, a trend for a statistically significant decrease in the D group, and no statistically significant change in the E group. Nonoverlapping 95% confidence intervals were also observed for the DE group. However, for all groups, the 95% PI for changes in non-HDL-C included zero (0). Changes in non-HDL-C were equivalent to −6.5%, −5.6% and 0.8%, respectively, for DE, D, and E groups. No outliers or heterogeneity were observed. In addition, no small-study effects were found as the funnel plot was generally symmetrical and no data points had to be imputed (Figure 2). When between-group changes in non-HDL-C were calculated, no statistically significant difference was observed (*Q*_*b*_ = 4.2, *P* = 0.13). No statistically significant or trend for a statistical association was found between changes in non-HDL-C and age, initial non-HDL-C, and changes in bod y weight (*P* > 0.10 for all). 

## 4. Discussion

 To the best of the authors' knowledge, this is the first meta-analytic study to examine the effects of D, E, and DE on changes in non-HDL-C in adult humans. The overall findings suggest that DE reduces non-HDL-C in adults, while there was a trend for statistically reductions in the D group. No statistically significant reductions were found for the E group. The observed changes in non-HDL-C for the DE and D groups were almost entirely the result of statistically significant decreases in TC and little or no change in HDL-C [8]. 

 The approximate 7% decrease observed in the DE group may be clinically important. A recent meta-analysis by Robinson et al. found that most lipid-modifying drugs used as monotherapy have an approximate one to one relationship between percent non-HDL-C lowering and reduction in coronary heart disease [27]. Assuming that the same benefits could be achieved as a result of the current interventions, this would result in an approximate 7% reduction in coronary heart disease in the DE groups and an approximate 6% reduction in the D group. The National Cholesterol Education Program currently recommends a target for non-HDL-C, that is, 30 mg/dL higher than the target for LDL-C [3]. Given the current findings, it appears that DE, and possibly D, may contribute to achieving that goal. 

 No statistically significant associations were found between changes in non-HDL-C and age, initial non-HDL-C and changes in body weight. While these results are interesting, they should be interpreted with caution since studies in a meta-analysis are not randomly assigned to predictors [28]. Therefore, these potential predictors should be tested in large, randomized controlled trials. 

 While the results of this study suggest that DE, and possibly D, reduce non-HDL-C in adults, they should be interpreted with respect to the following. First, the 95% PI included zero (0) for all groups. This suggests that reductions in non-HDL-C may not occur in every setting. Second, given the small number of studies included as well as missing data, a determination of the optimal diet and dose of aerobic exercise needed to reduce non-HDL-C in adults could not be elucidated. Given the need to determine such, it is suggested that future randomized controlled trials address this issue. Third, because none of the studies reported non-HDL-C, variances were estimated based on the data reported for TC and HDL-C. This could have possibly led to results that are different than if the original variance statistics had been available. Given the former, it is strongly suggested that future studies report non-HDL-C, including the variance statistics for such. Fourth, the results of this study, like most studies, should not be generalized beyond the characteristics of the participants included.

## 5. Conclusions

Combined diet and aerobic exercise may reduce non-HDL-C among adults in some settings. However, future randomized controlled trials are needed before any final recommendations can be made.

## Figures and Tables

**Table 1:** Baseline values for non-HDL-C (mg/dL).

Group	Studies^#^	Groups^#^	Participants^#^	$\overset{-}{x}$ ± SD	Range	Median
Diet	6	7	207	169.0 ± 20.3	136–192	167
Exercise	6	7	192	160.9 ± 29.3	121–192	167
Diet and exercise	6	7	194	168.9 ± 26.4	130–201	162
Control	6	7	195	162.7 ± 24.3	135–192	159

^#^Number; $\overset{-}{x}\pm \text{SD}$, mean ± standard deviation; to convert from mg/dL to mmol, divide by 38.67.

**Table 2:** Changes in non-HDL-C (mg/dL).

Variable	Studies^#^	Participants^#^ (I + C)	ES^#^	$\overset{-}{x}$ (95% CI) (mg/dL)	*P *	*Q*(*P*)	*I* ^2^ (%)	95% PI
Diet	6	402	7	−8.5 (−18.6, 1.6)	0.10**	0.8 (0.99)	0	−21.7, 4.8
Exercise	6	389	7	3.0 (−7.1, 13.1)	0.60	0.8 (0.99)	0	−10.3, 16.3
Diet + Exercise	6	387	7	−11.1 (−21.7, −0.6)	0.04*	2.4 (0.88)	0	−24.4, 2.1

^#^Number; ES: effect sizes; $\overset{-}{x}$ (95% CI), mean ± 95% confidence intervals; *P*: alpha value for changes in non-HDL-C; *Q*(*P*): Cochran's *Q* statistic and associated alpha value; *I* ^2^ (%): percentage of inconsistency; 95% PI: 95% prediction intervals; I + C: intervention + control; *statistically significant at *P* ≤ 0.05; **trend (>0.05 to ≤0.10) for statistical significance; To convert changes in mg/dL to mmol, divide by 38.67.
